# Perioperative Coagulation Dynamics Assessed by ROTEM and Conventional Assays: A Prospective Longitudinal Study with Focus on Fibrinogen in Major Non-Cardiac Surgery

**DOI:** 10.3390/jcm15114264

**Published:** 2026-05-31

**Authors:** Michal Hosala, Denisa Osinova, Matej Sukenik, Janka Hosalova Matisova, Tomas Fecko, Jana Sendreyova, Kristina Maria Belakova, Miroslava Drotarova, Monika Brunclikova, Sohaib Mukhtar Agouba, Veronika Voskova Gemelova, Tomas Simurda

**Affiliations:** 1 Department of General, Visceral and Transplant Surgery, Comenius University in Bratislava, Jessenius Faculty of Medicine and University Hospital Martin, 03601 Martin, Slovakia; michal.hosala@uniba.sk; 2 Department of Anesthesiology and Intensive Medicine, Comenius University in Bratislava, Jessenius Faculty of Medicine and University Hospital Martin, 03601 Martin, Slovakia; mato.sukenik@gmail.com (M.S.); hosalovajanka@gmail.com (J.H.M.); fecko.tf@gmail.com (T.F.); janacveckova@yahoo.com (J.S.); 3 Department of Hematology and Transfusiology, National Centre of Hemostasis and Thrombosis, Comenius University in Bratislava, Jessenius Faculty of Medicine and University Hospital Martin, 036 01 Martin, Slovakia; kristinabelakova@gmail.com (K.M.B.); miroslava.sterankova11@gmail.com (M.D.); simkovamonika@gmail.com (M.B.); sohaiib@yahoo.com (S.M.A.); veronika.gemelova@gmail.com (V.V.G.)

**Keywords:** rotational thromboelastometry, fibrinogen, FIBTEM, perioperative hemostasis, viscoelastic testing, coagulation dynamics

## Abstract

**Background:** Perioperative assessment of hemostasis remains challenging, particularly in non-cardiac surgery, where the incidence of clinically significant bleeding is relatively low. Viscoelastic testing enables real-time evaluation of whole-blood coagulation, but its perioperative dynamics and agreement with conventional assays require further characterization. **Methods:** In this prospective single-center observational study, 53 patients undergoing major non-cardiac surgery were included in the final analysis. Hemostatic parameters were assessed preoperatively, intraoperatively at two-hour intervals, and 24 h postoperatively using ROTEM (EXTEM, INTEM, and FIBTEM) and conventional coagulation assays. Longitudinal changes and correlations between fibrinogen concentration according to Clauss and ROTEM-derived parameters were analyzed. **Results:** Fifty-three patients undergoing major hepatopancreatobiliary surgery completed longitudinal perioperative monitoring. Pancreaticoduodenectomy and liver resection accounted for 60.7% and 39.3% of procedures, respectively. Serial perioperative ROTEM and laboratory assessments demonstrated time-dependent changes in coagulation parameters, including decreased intraoperative fibrinogen and antithrombin III levels and shortening of INTEM clotting time. Clinically significant bleeding was infrequent, with severe intraoperative bleeding observed in 7.4% of patients. Strong correlations were confirmed between fibrinogen concentration according to Clauss and FIBTEM-derived parameters, particularly FIBTEM MCF (r = 0.811, *p* < 0.001), whereas the correlation with FIBTEM A10 was moderate (r = 0.574, *p* < 0.001). No significant association was observed between preoperative fibrinogen concentration and perioperative blood loss. **Conclusions:** This study provides a longitudinal characterization of perioperative coagulation dynamics and demonstrates strong agreement between fibrinogen concentration according to Clauss and ROTEM-derived parameters. The findings are primarily descriptive and highlight the methodological consistency between viscoelastic and conventional fibrinogen assessment. Further studies are required to determine their clinical relevance in perioperative management. These findings suggest that ROTEM-derived parameters may provide a rapid functional estimate of fibrinogen status in the perioperative setting, potentially supporting clinical assessment, although their role in guiding management requires further validation.

## 1. Introduction

Perioperative bleeding remains a significant cause of morbidity and mortality during major surgery and represents an important challenge for anesthesiologists and perioperative care teams. Accurate and timely assessment of coagulation status is essential for guiding transfusion therapy and preventing complications associated with both bleeding and unnecessary administration of blood products [1,2]. Conventional laboratory coagulation assays such as prothrombin time (PT), activated partial thromboplastin time (aPTT), thrombin time (TT), and plasma fibrinogen concentration are commonly used to evaluate hemostasis. However, these tests have several limitations in the perioperative setting, including relatively long turnaround time and limited ability to reflect the dynamic and complex interactions of the coagulation cascade in whole blood [3,4]. As a result, they may not adequately capture real-time changes in coagulation during major surgical procedures.

Viscoelastic hemostatic assays, including rotational thromboelastometry (ROTEM), have been increasingly used for point-of-care assessment of coagulation. ROTEM provides rapid evaluation of clot initiation, clot formation, clot strength, and fibrinolysis, offering a comprehensive assessment of hemostatic function in whole blood [5,6]. In recent years, ROTEM-guided transfusion strategies have gained increasing attention in perioperative medicine and have been shown to reduce blood product consumption in several clinical settings [5,7]. Recent prospective studies in major surgery settings further support the role of ROTEM in detecting time-dependent perioperative alterations in coagulation and viscoelastic clot formation parameters and identifying patients at risk of bleeding [8].

Fibrinogen plays a pivotal role in clot formation and is considered one of the most important determinants of clot firmness. Low fibrinogen concentrations are associated with increased risk of perioperative bleeding, particularly during major surgery [9]. The fibrinogen concentration according to Clauss is widely regarded as the standard laboratory method for measuring plasma fibrinogen concentration. However, ROTEM-based parameters such as FIBTEM amplitude at 10 min (A10) and maximum clot firmness (MCF) provide a rapid estimate of functional fibrinogen contribution to clot strength [10].

Previous studies have suggested that the relationship between preoperative fibrinogen concentrations and perioperative bleeding may be complex. For example, a study by Mion et al. reported a U-shaped association between fibrinogen activity and severe perioperative bleeding in patients undergoing cardiac surgery, indicating that both low and high fibrinogen concentrations may be associated with increased bleeding risk [11]. However, data regarding this relationship in patients undergoing major non-cardiac surgery remain limited.

Although correlations between Clauss fibrinogen concentration and ROTEM-derived FIBTEM parameters have been previously described, less is known about the temporal stability of these relationships during prolonged major non-cardiac surgery with evolving perioperative hemostatic changes. In addition, prospective longitudinal data with repeated intraoperative measurements in hepatopancreatobiliary surgery remain limited. Such procedures are associated with substantial physiological stress, inflammatory activation, fluid shifts, and variable bleeding risk, all of which may dynamically influence clot formation and fibrinogen-related clot firmness.

Therefore, the aim of this prospective observational study was to longitudinally characterize perioperative coagulation dynamics in patients undergoing major non-cardiac surgery using both ROTEM analysis and conventional coagulation assays. The study further aimed to evaluate the relationship between plasma fibrinogen concentration measured by the Clauss method and ROTEM-derived FIBTEM parameters, as well as their association with perioperative bleeding outcomes.

## 2. Materials and Methods

### 2.1. Study Design and Setting

This was a prospective, single-center, investigator-initiated observational study conducted in routine clinical practice. As the study did not evaluate the efficacy or safety of a medicinal product or medical device, and all procedures followed standard clinical practice, registration in a clinical trial registry was not required according to applicable regulations.

### 2.2. Study Population

Adult patients scheduled for major non-cardiac surgery under general or combined anesthesia were considered eligible for inclusion. Although several procedure types were prespecified in the study protocol, only pancreaticoduodenectomy (Whipple procedure) (60.7%) and liver resection (39.3%) were ultimately represented in the analyzed cohort. Consecutive patients fulfilling the eligibility criteria were invited to participate.

### 2.3. Inclusion and Exclusion Criteria

Patients were excluded if they had known hematological diseases or congenital coagulation disorders or if they were receiving anticoagulant or antiplatelet therapy with an ongoing pharmacological effect at the time of surgery.

### 2.4. Data Collection

Baseline patient characteristics, including age, sex, body mass index, smoking status, comorbidities, and American Society of Anesthesiologists (ASA) physical status classification, were recorded. Preoperative laboratory values collected within 24 h prior to surgery included hemoglobin, hematocrit, platelet count, leukocyte count, PT, aPTT, TT, fibrinogen concentration measured by the Clauss method, antithrombin III, and C-reactive protein.

### 2.5. Hemostasis Monitoring

Perioperative coagulation status was assessed using rotational thromboelastometry (ROTEM) and standard laboratory coagulation assays. ROTEM analyses were performed using the ROTEM^®^ delta system (Tem International GmbH, Munich, Germany). Conventional coagulation assays were performed under standard laboratory conditions at 37 °C according to institutional laboratory practice. ROTEM analyses were performed using standard manufacturer operating conditions and were not adjusted to individual patient core temperature. The evaluated parameters included EXTEM, INTEM, and FIBTEM assays, with measurement of clotting time (CT), clot formation time (CFT), A10, MCF, and maximum lysis (ML). Hemostatic parameters were recorded at predefined time points: at the start of surgery (baseline), every two hours during the surgical procedure, and 24 h postoperatively. Conventional coagulation parameters (PT, aPTT, TT, fibrinogen concentration, and antithrombin III) were assessed at the same time points.

### 2.6. Perioperative Outcome Measures

Perioperative clinical data included total blood loss during surgery and within the first 24 h after surgery, severity of bleeding, duration of the surgical procedure, transfusion requirements (erythrocytes, fresh frozen plasma, and platelets), and administration of hemostatic therapies, including fibrinogen concentrate, prothrombin complex concentrate, recombinant activated factor VII, and tranexamic acid. The length of stay in the intensive care unit and total duration of hospitalization were also recorded.

### 2.7. Definition of Bleeding Severity and Transfusion Criteria

Bleeding severity was categorized according to institutional clinical criteria based on estimated blood loss and clinical impact, consistent with the predefined study data collection. Patients were stratified into categories ranging from minimal bleeding (no intervention required) to severe bleeding (requiring transfusion support, hemodynamic stabilization, or surgical reintervention). Transfusion and hemostatic therapy were administered according to standard institutional practice and at the discretion of the treating anesthesiologist. Indications for transfusion included hemoglobin thresholds, clinical signs of hypoperfusion, and ongoing bleeding. Administration of plasma, fibrinogen concentrate, and tranexamic acid was guided by clinical judgment, with consideration of laboratory and ROTEM findings when available.

### 2.8. Timing of Interventions Relative to ROTEM Measurements

All transfusions and hemostatic interventions were prospectively recorded in relation to predefined sampling time points (baseline, intraoperative measurements at two-hour intervals, and 24 h postoperative assessment), allowing for contextual interpretation of coagulation parameters with respect to potential treatment effects.

### 2.9. Statistical Analysis

Statistical analyses were primarily descriptive, reflecting the observational design of the study and the predefined statistical analysis plan. Continuous variables were summarized using mean ± standard deviation or median (interquartile range), as appropriate, and categorical variables as counts and percentages. Longitudinal changes in coagulation parameters across predefined perioperative time points were evaluated descriptively using 95% confidence intervals for the mean. Given the relatively small sample size, decreasing number of observations at later intraoperative time points (e.g., *n* = 10 at 6 h), and low number of clinically relevant bleeding events, no formal repeated-measures modeling (e.g., mixed-effects models) was applied. Formal hypothesis testing for longitudinal comparisons was not the primary objective of the analysis. Time-dependent changes were therefore interpreted descriptively using mean values and 95% confidence intervals. Correlations between fibrinogen concentration according to Clauss and ROTEM-derived parameters were assessed using Pearson’s or Spearman’s correlation coefficients, depending on data distribution. No adjustment for potential confounders, including transfusion therapy and administration of fibrinogen concentrate or tranexamic acid, was performed due to their low frequency and limited statistical power for subgroup analyses. The analysis was therefore considered exploratory and hypothesis-generating rather than confirmatory.

## 3. Results

A total of 61 patients were enrolled in the study. Eight patients were excluded from the longitudinal analysis because surgery was aborted due to intraoperative inoperability detected after enrollment. Therefore, 53 patients were included in the final perioperative analysis. Baseline characteristics are reported for all 61 enrolled patients (Table 1), whereas perioperative analyses were performed in the 53 patients who completed the surgical procedure. The study population included 32 males (52.5%) and 29 females (47.5%), with a mean age of 62.7 ± 12.4 years and a mean body mass index of 28.5 ± 6.8 kg/m^2^. Active malignancy and arterial hypertension were present in 57.4% of patients, diabetes mellitus in 31.1%, and anemia in 14.8%. According to the American Society of Anesthesiologists (ASA) classification, 31.1% of patients were classified as ASA II, 65.6% as ASA III, and 3.3% as ASA IV.

### 3.1. Surgical Characteristics

The most frequently performed procedures were duodenopancreatectomy (60.7%) and liver resection (39.3%). The duration of surgery varied widely, ranging from 45 to 525 min, with a mean duration of 296.8 ± 109.5 min.

Mean intraoperative body temperature showed a slight increase during surgery, from 36.30 ± 0.35 °C at the beginning of the procedure to 36.49 ± 0.36 °C after two hours and 36.71 ± 0.40 °C after four hours, reflecting active intraoperative temperature management.

### 3.2. Perioperative Hemostasis Parameters

Perioperative coagulation parameters were monitored using both ROTEM and conventional laboratory tests at baseline, every two hours during surgery, and 24 h postoperatively. Mean values of selected ROTEM and laboratory coagulation parameters across perioperative time points are presented in Table 2.

Perioperative trends were observed in several coagulation parameters across the monitored time points. Clotting time in the INTEM assay decreased during surgery, reaching lower values at six hours compared with baseline and postoperative measurements. Similarly, aPTT values showed a decreasing trend during the intraoperative period and remained lower postoperatively compared with baseline values.

**Figure 1:**
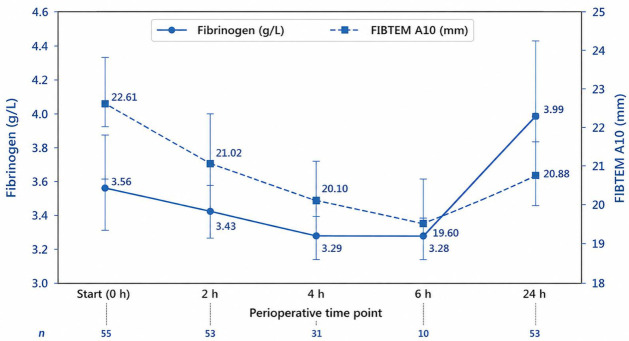
Perioperative trend of fibrinogen concentration and FIBTEM A10 during major non-cardiac surgery. Values are presented as mean ± 95% confidence intervals. *n* denotes the number of patients with measurements at each perioperative time point for fibrinogen and FIBTEM A10.

Fibrinogen concentrations decreased during the intraoperative period, reaching lower values at four hours of surgery compared with postoperative measurements. These perioperative trends are illustrated in Figure 1. Antithrombin III activity also decreased during surgery compared with baseline concentrations.

Comparison of preoperative and postoperative laboratory parameters demonstrated lower postoperative values of hemoglobin (130.3 g/L vs. 115.9 g/L), hematocrit (0.39 vs. 0.34), and aPTT, whereas leukocyte count and C-reactive protein were higher postoperatively. These differences were identified descriptively based on non-overlapping 95% confidence intervals.

### 3.3. Blood Loss and Transfusion Therapy

Mean total blood loss during surgery was 512 ± 381 mL (median 400 mL), while mean postoperative blood loss within the first 24 h was 391 ± 409 mL (median 260 mL).

Most patients experienced minimal perioperative bleeding. Severe bleeding occurred in 7.4% of patients during surgery and 5.7% postoperatively, while massive bleeding was observed in one patient (1.9%) after surgery.

Transfusion of erythrocytes and fresh frozen plasma was required in 20.4% and 16.7% of patients during surgery, respectively. Postoperatively, erythrocyte transfusion was administered in 5.7% of patients and plasma transfusion in 11.3%. No platelet transfusions were recorded.

Hemostatic therapy during surgery was required in a limited number of patients. Fibrinogen concentrate was administered to three patients at four hours and to one patient at six hours of surgery, while tranexamic acid was administered to four patients in total.

### 3.4. Clinical Outcomes

The mean length of stay in the intensive care unit was 4.7 ± 3.6 days, and the mean total duration of hospitalization was 12.7 ± 7.5 days. Reoperation within 24 h was required in one patient (2.0%).

### 3.5. Impact of Hemostatic Therapy on Coagulation Parameters

Hemostatic interventions were infrequent. Fibrinogen concentrate was administered to four patients (three at 4 h and one at 6 h intraoperatively), while tranexamic acid was administered to four patients at defined intraoperative time points. No administration of prothrombin complex concentrate or recombinant activated factor VII was observed.

Given the low frequency of interventions, changes in ROTEM parameters are more likely to reflect physiological perioperative coagulation dynamics rather than treatment effects at the population level. However, interpretation at the individual level should consider potential therapy-related influences.

Therefore, no subgroup analysis stratified by hemostatic intervention was performed.

### 3.6. Correlation Analyses

**Figure 2:**
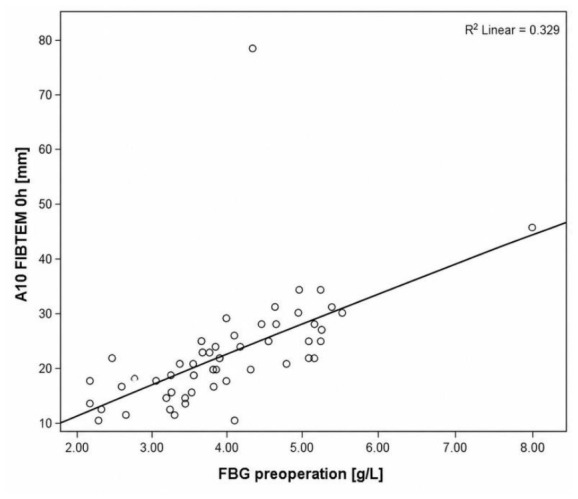
Correlation between fibrinogen concentration according to Clauss and FIBTEM A10 before surgery.

**Figure 3:**
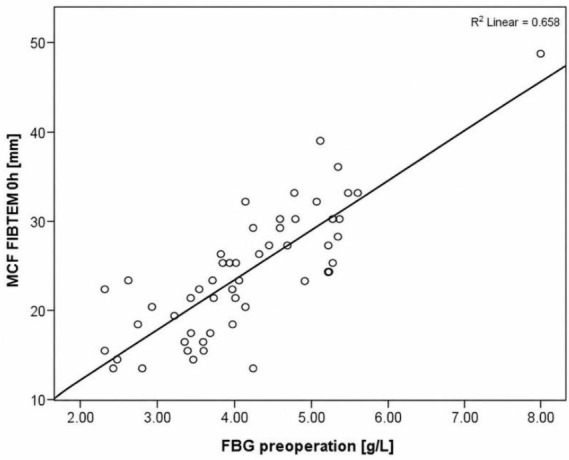
Correlation between fibrinogen concentration according to Clauss and FIBTEM MCF before surgery.

A significant correlation was observed between fibrinogen concentration measured by the Clauss method and ROTEM-derived FIBTEM parameters (A10: r = 0.574, *p* < 0.001; MCF: r = 0.811, *p* < 0.001) (Figure 2 and Figure 3).

No significant correlation was found between preoperative fibrinogen concentration and total perioperative blood loss or severity of bleeding. Additionally, no significant association was observed between total intraoperative blood loss and duration of ICU stay or overall length of hospitalization.

## 4. Discussion

The present prospective observational study evaluated perioperative changes in hemostasis using ROTEM and conventional laboratory coagulation assays in patients undergoing major non-cardiac surgery. A key aspect of the present study is the low incidence of clinically relevant bleeding events, which inherently limits the ability to draw outcome-based or predictive conclusions. Under such conditions, the absence of association between coagulation parameters and bleeding outcomes should not be interpreted as evidence of no relationship but rather as a reflection of limited statistical power. Accordingly, the findings should be interpreted primarily as a detailed physiological and methodological characterization of perioperative coagulation dynamics rather than as evidence supporting predictive clinical utility. Time-dependent longitudinal perioperative alterations in coagulation and viscoelastic clot formation parameters measured across predefined intraoperative and postoperative sampling intervals were observed throughout the study period. dynamic perioperative coagulation changes.

Perioperative bleeding is a well-recognized complication of major surgery and remains an important contributor to morbidity, prolonged hospitalization, and increased healthcare costs [1,2]. Accurate monitoring of coagulation status is therefore essential to guide transfusion therapy and optimize perioperative hemostatic management. Conventional coagulation assays such as PT and aPTT are widely used but have important limitations, including delayed turnaround time and limited ability to reflect the complex interactions of whole-blood coagulation during active bleeding [3].

Viscoelastic assays such as ROTEM provide a dynamic assessment of clot formation, clot strength, and fibrinolysis and are increasingly used in perioperative medicine [5,12]. Several studies have demonstrated that ROTEM-guided transfusion algorithms may reduce blood product consumption and improve hemostatic management in surgical and trauma settings [7]. In the present study, ROTEM monitoring allowed for a detailed assessment of perioperative changes in coagulation parameters, demonstrating measurable alterations in clotting dynamics during surgery.

Fibrinogen plays a central role in clot formation and is considered a key determinant of clot strength. Hypofibrinogenemia is frequently associated with increased bleeding risk during major surgery [9]. Our results demonstrated a significant correlation between fibrinogen concentration according to Clauss and FIBTEM parameters (A10 and MCF), supporting the use of FIBTEM as a rapid point-of-care estimate of fibrinogen-related clot strength. Similar correlations between fibrinogen concentration according to Clauss and FIBTEM parameters have been reported in previous studies evaluating perioperative coagulation monitoring [9,13,14]. Recent reviews highlight the increasing clinical relevance of viscoelastic testing for rapid assessment of fibrinogen-related clot strength in various clinical settings [15]. Similar correlations between fibrinogen concentration and FIBTEM-derived parameters have also been reported in more recent studies, supporting the role of FIBTEM as a rapid functional surrogate of fibrinogen status [16]. Importantly, the present findings should not be interpreted as demonstrating a novel biological relationship between fibrinogen concentration according to Clauss and FIBTEM-derived parameters, as such correlations have been consistently reported in previous perioperative and critical care studies. Rather, the present study extends existing evidence by prospectively characterizing the temporal behavior of fibrinogen-related clot firmness during major hepatopancreatobiliary surgery using repeated intraoperative measurements under dynamically changing perioperative conditions. The longitudinal design enabled evaluation of coagulation trajectories during prolonged surgery, including progressive intraoperative decreases in fibrinogen concentration and antithrombin III activity, accompanied by relatively stable FIBTEM clot firmness parameters despite ongoing surgical stress and fluid administration. The stronger correlation observed between fibrinogen concentration according to Clauss and FIBTEM MCF compared with A10 likely reflects physiological differences between these parameters. MCF represents the fully developed final clot structure and maximal fibrin-based clot strength, whereas A10 reflects an earlier stage of clot propagation that may be more susceptible to transient perioperative variability, hemodilution, and changes in clot kinetics. Nevertheless, despite the lower correlation coefficient, A10 may still retain clinical utility as a rapid intraoperative parameter because it becomes available substantially earlier during active bleeding management. dynamic perioperative changes.

The strong agreement between fibrinogen concentration according to Clauss and FIBTEM parameters observed in this study is consistent with previous reports and supports the use of viscoelastic testing as a rapid functional assessment of fibrinogen contribution to clot strength. However, the present study was not designed to evaluate clinical decision-making or outcome-based benefits of ROTEM-guided management, and such conclusions cannot be drawn from these data. It should be noted that Clauss fibrinogen and FIBTEM parameters reflect different aspects of fibrinogen biology and may provide complementary rather than interchangeable information in clinical practice [17].

These findings suggest that ROTEM-derived parameters may serve as a practical point-of-care surrogate for fibrinogen assessment, potentially facilitating timely clinical evaluation in the perioperative setting, although their impact on decision-making and outcomes requires further investigation.

No significant association was observed between preoperative fibrinogen concentration and perioperative bleeding outcomes. However, this finding should be interpreted with caution, given the low number of clinically relevant bleeding events, which substantially limits statistical power for outcome-based analyses. The longitudinal design with repeated perioperative measurements represents a major strength of this study, allowing for characterization of time-dependent changes in coagulation parameters. Although advanced longitudinal modeling techniques (such as mixed-effects models) were not applied, the repeated-measures approach provides valuable descriptive insight into intraoperative coagulation trajectories. These findings may inform the design of future studies focusing on dynamic, time-dependent associations between coagulation parameters and clinical outcomes. Previous research has suggested that the relationship between fibrinogen concentrations and perioperative bleeding may be more complex. For example, Mion et al. reported a U-shaped relationship between fibrinogen activity and severe perioperative bleeding in patients undergoing cardiac surgery, suggesting that both low and high fibrinogen concentrations may be associated with increased bleeding risk [11]. Further research is therefore needed to better define optimal perioperative fibrinogen thresholds in non-cardiac surgical populations.

Exploratory subgroup analyses demonstrated numerically higher median fibrinogen concentrations and blood loss in oncology patients compared with non-oncology patients, although statistically significant differences were not observed. These observations may warrant further investigation in larger oncologic surgical cohorts. Another important observation in this study was the relatively low requirement for hemostatic therapy and transfusion support. Most patients experienced minimal perioperative bleeding, and only a small proportion required administration of fibrinogen concentrate or tranexamic acid. The low requirement for transfusion and hemostatic therapy was consistent with the low incidence of clinically significant bleeding in this cohort.

Several limitations of this study should be acknowledged. First, this was a single-center observational study with a relatively small sample size, which may limit the generalizability of the findings. Second, the number of patients experiencing severe bleeding was low, reducing the statistical power to detect associations between coagulation parameters and bleeding outcomes. The decreasing number of observations at later intraoperative time points, particularly at 6 h, limits the interpretation of apparent temporal trends and increases susceptibility to sampling variability. The low frequency of clinically relevant bleeding events and hemostatic interventions limited the feasibility of meaningful multivariable analyses in this cohort. In addition, the study population included several types of major surgical procedures with potentially different bleeding risks. Finally, the study population mainly consisted of patients undergoing hepatobiliary and pancreatic surgery, which may limit the extrapolation of the results to other surgical populations. Robotic-assisted procedures were not systematically recorded in the study dataset and therefore could not be reliably analyzed. Detailed oncologic treatment history, including neoadjuvant chemotherapy and radiotherapy, was not prospectively collected and therefore could not be included in subgroup analyses. Furthermore, the heterogeneity of surgical procedures and the lack of standardized transfusion triggers may have introduced additional variability in clinical outcomes. Despite these limitations, the present study provides valuable prospective data on perioperative hemostasis monitoring using both ROTEM and conventional laboratory tests in major non-cardiac surgery. These findings support the methodological agreement between viscoelastic and conventional fibrinogen assessment, while further research is required to determine their clinical applicability in perioperative decision-making.

Future studies with larger patient cohorts and multicenter designs are needed to further evaluate the predictive value of viscoelastic parameters for perioperative bleeding and to optimize ROTEM-guided hemostatic management strategies.

## 5. Conclusions

This prospective longitudinal study provides a detailed characterization of perioperative coagulation trajectories during major hepatopancreatobiliary surgery using combined ROTEM and conventional coagulation testing. The study confirms strong methodological agreement between Clauss fibrinogen concentration and FIBTEM-derived clot firmness parameters across dynamically changing perioperative conditions. Although the low incidence of clinically relevant bleeding limited outcome-based analyses, the findings provide important descriptive insight into temporal perioperative hemostatic alterations and may support future studies evaluating longitudinal viscoelastic monitoring in major non-cardiac surgery.

## Figures and Tables

**Table 1:** Baseline characteristics of the study population.

	Mean ± SD
Age (years)	62.66 ± 12.36
Height (cm)	171.16 ± 8.22
Weight (kg)	82.82 ± 17.31
Body mass index (kg/m^2^)	28.46 ± 6.80
	*n* (%)
Male sex	32 (52.5)
Female sex	29 (47.5)
Arterial hypertension	35 (57.4)
Diabetes mellitus	19 (31.1)
Ischemic heart disease	12 (19.7)
Anemia	9 (14.8)
Active malignancy	35 (57.4)
Smoking	12 (19.7)
	*n* (%)
ASA II	19 (31.1)
ASA III	40 (65.6)
ASA IV	2 (3.3)

Abbreviations: ASA—American Society of Anesthesiologists, SD—standard deviation, *n*—number of patients.

**Table 2:** Selected perioperative ROTEM and laboratory coagulation parameters.

Parameter	Reference Range	0 h Mean ± SD (*n* = 55)	2 h Mean ± SD (*n* = 53)	4 h Mean ± SD (*n* = 31)	6 h Mean ± SD (*n* = 10)	24 h Mean ± SD (*n* = 53)
CT EXTEM (s)	38–79	72.26 ± 17.89	70.79 ± 12.81	65.90 ± 9.33	69.10 ± 8.31	73.72 ± 12.09
A10 EXTEM (mm)	43–65	60.26 ± 7.98	59.85 ± 8.00	60.16 ± 7.64	58.90 ± 9.49	60.14 ± 6.20
MCF EXTEM (mm)	50–72	67.30 ± 6.11	67.35 ± 6.32	68.29 ± 5.68	66.80 ± 6.76	66.70 ± 4.75
A10 FIBTEM (mm)	7–23	22.61 ± 10.36	21.02 ± 7.26	20.10 ± 6.72	19.60 ± 8.06	20.88 ± 5.50
MCF FIBTEM (mm)	9–25	23.67 ± 7.53	23.37 ± 7.94	22.68 ± 7.45	22.20 ± 9.22	23.80 ± 6.15
CT INTEM (s)	100–240	206.06 ± 41.82	193.90 ± 59.70	189.77 ± 45.95	168.90 ± 19.97	195.36 ± 38.06
APTT (s)	25–35	31.39 ± 3.96	29.80 ± 8.84	28.23 ± 7.06	27.35 ± 2.25	29.05 ± 3.33
Fibrinogen (g/L)	2.0–4.0	3.56 ± 0.98	3.43 ± 1.02	3.29 ± 1.04	3.28 ± 0.79	3.99 ± 1.06

Abbreviations: CT—clotting time; A10—amplitude at 10 min; MCF—maximum clot firmness; EXTEM—extrinsic activation assay; INTEM—intrinsic activation assay; FIBTEM—fibrin-based clot formation assay; *n*—number of available observations with valid measurements at the respective perioperative time point.

## Data Availability

The datasets used and analyzed during the current study are available from the corresponding author upon reasonable request.
